# Generation of genome-edited dogs by somatic cell nuclear transfer

**DOI:** 10.1186/s12896-022-00749-3

**Published:** 2022-07-13

**Authors:** Dong-Ern Kim, Ji-Hye Lee, Kuk-Bin Ji, Kang-Sun Park, Tae-Young Kil, Okjae Koo, Min-Kyu Kim

**Affiliations:** 1 Laboratory of Animal Reproduction and Physiology, Department of Animal Science and Biotechnology, College of Agriculture and Life Science, Chungnam National University, Daejeon, 34134 Korea; 2 MK biotech Inc., Daejeon, 34134 Korea; 3 Department of Social Welfare, Joongbu University, Geumsan, 32713 Korea; 4 ToolGen Inc., Seoul, 08501 Korea

**Keywords:** Genome-edited dog, CRISPR-Cas9, Somatic nuclear transfer, DJ-1 knock out

## Abstract

**Background:**

Canine cloning technology based on somatic cell nuclear transfer (SCNT) combined with genome-editing tools such as CRISPR-Cas9 can be used to correct pathogenic mutations in purebred dogs or to generate animal models of disease.

**Results:**

We constructed a CRISPR-Cas9 vector targeting canine DJ-1. Genome-edited canine fibroblasts were established using vector transfection and antibiotic selection. We performed canine SCNT using genome-edited fibroblasts and successfully generated two genome-edited dogs. Both genome-edited dogs had insertion-deletion mutations at the target locus, and DJ-1 expression was either downregulated or completely repressed.

**Conclusion:**

SCNT successfully produced genome-edited dogs by using the CRISPR-Cas9 system for the first time.

**Supplementary Information:**

The online version contains supplementary material available at 10.1186/s12896-022-00749-3.

## Background

Canine cloning technology based on somatic cell nuclear transfer (SCNT) has been used for various purposes since Snuppy was first cloned in 2005 [1]. It has been used not only for pet cloning [2] but also for propagating elite working dogs, including sniffing dogs [3] and rescue dogs [4]. This technology has also been used to preserve rare canine breeds [5, 6] and endangered canid species such as wolves [7, 8]. Canine cloning technology provides a platform for generating genetically engineered dogs. Previous studies have shown that transgenic dogs cloned from transgenic canine fibroblasts express transgenes [9] and can stably transfer the integrated transgene to the next generation [10]. These results are particularly interesting because this technology may be useful for producing dogs as models of human diseases [11].

The recent development of genome-editing tools, including CRISPR-Cas9 technology, has dramatically changed the field of animal genetic engineering. Unlike previous transgenic technologies, which tend to integrate exogenous gene sequences into genomes, genome-editing tools recognize specific target sequences in the genome, induce double-strand breaks, and efficiently edit genomic sequences as needed [12]. This new technology has significantly changed the approaches used for the application of genetically engineered animals. Scientists and companies have attempted to use this technology to improve the genetic traits of livestock and to generate disease models. In previous studies, researchers were able to produce disease-resistant pigs [13] and cows [14] and enhance the productivity of pigs [15] and cashmere goats [16]. This technology has also been used to improve animal welfare by producing hornless dairy cattle [17].

However, in the canine research field, only two cases have been reported in which genome-editing tools, specifically CRISPR-Cas9, were used to produce genetically engineered dogs [18, 19]. However, none of these studies used SCNT-based canine cloning technology. The advantage of SCNT-based canine genome-editing is that it can maintain the breed, genotypic background, and phenotype, except for the genome-edited target loci of donor animals. In particular, this technology can be used to recover pathogenic mutations in purebred dogs or to generate inbred animal models to study diseases.

The current study was performed to establish procedure for SCNT-based canine genome editing. In particular, the DJ-1 gene was selected as the genome-editing target. DJ-1 is a multifunctional protein that is expressed in almost all cells and tissues [20]. It was originally identified as an oncogene [21] and is now known to be related to various neurodegenerative disorders, including Parkinson’s disease (PD), Alzheimer’s disease, and ischemic stroke [22]. Accumulating evidence indicates that DJ-1 is a useful therapeutic target for immune, inflammatory [23], and ocular diseases [24]. DJ-1-deficient mice have been generated to evaluate the role and function of this gene, but their disease phenotypes are not similar to those of humans [25]. In contrast to knockout (KO) mouse models, DJ-1-deficient rat models show a disease phenotype similar to that of human patients [26, 27]. Therefore, larger animal models, such as dogs, may provide more accurate data for studying DJ-1-related diseases in humans. In the same context, certain dog breeds appear to be spontaneous models for PD, whereas rodent species are rare. Canine multiple system degeneration (CMSD) is a fatal heritable movement disorder [28, 29]. It progresses to cerebellar ataxia and postural instability, and histological features include an olivary and caudate nuclei, and substantia nigra [28]. It is associated with the pathogenesis of PD and the CMSD locus contains the parkin gene PARK2. Mutations in PARK2 are known to cause familial PD with clinical and pathological similarities to CMSD [29]. Additionally, beagles aged 5 and 9 years have been demonstrated to exhibit cognitive impairment in humans aged 40–60 years, making them an ideal aging model [30]. This feature is an advantage of using dogs as models for neurological disorders since the dog's brain is subjected to stress similar to that of humans [31]. Therefore, the current study was performed to generate neurologic disease model dog.

## Results and discussion

We successfully produced CRISPR-Cas9-mediated genome-edited dogs using the SCNT technology. We targeted DJ-1 in the canine genome and confirmed an insertion-deletion mutation at the target locus of genome-edited dogs, whereas no off-targets were found at any potential off-target locus. We also confirmed that the expression of DJ-1 was downregulated or completely repressed in genome-edited dogs.

**Fig. 1:**
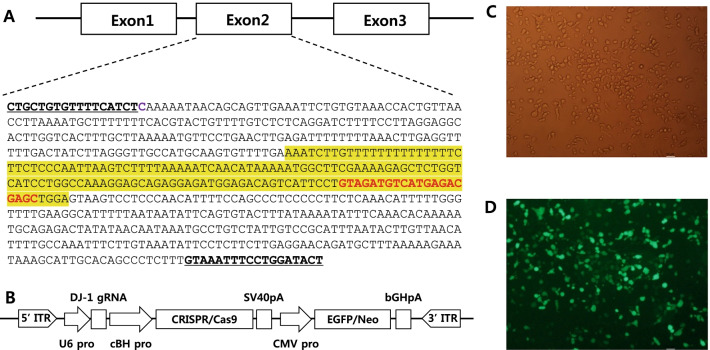
Design and expression of CRISPR-Cas9 vector. **A** Guide RNA (gRNA, red letters) sequence selected from exon 2 of canine DJ-1 (highlighted in yellow). The bold, underlined letters at the front and end of the sequences were primer sets used for sequencing analysis. **B** Schemes for CRISPR-Cas9 vector construct used in the study. Expression of the vector in transfected cells was confirmed based on EGFP expression evaluated using **C** bright view microscopy and **D** ultraviolet light

**Fig. 2:**
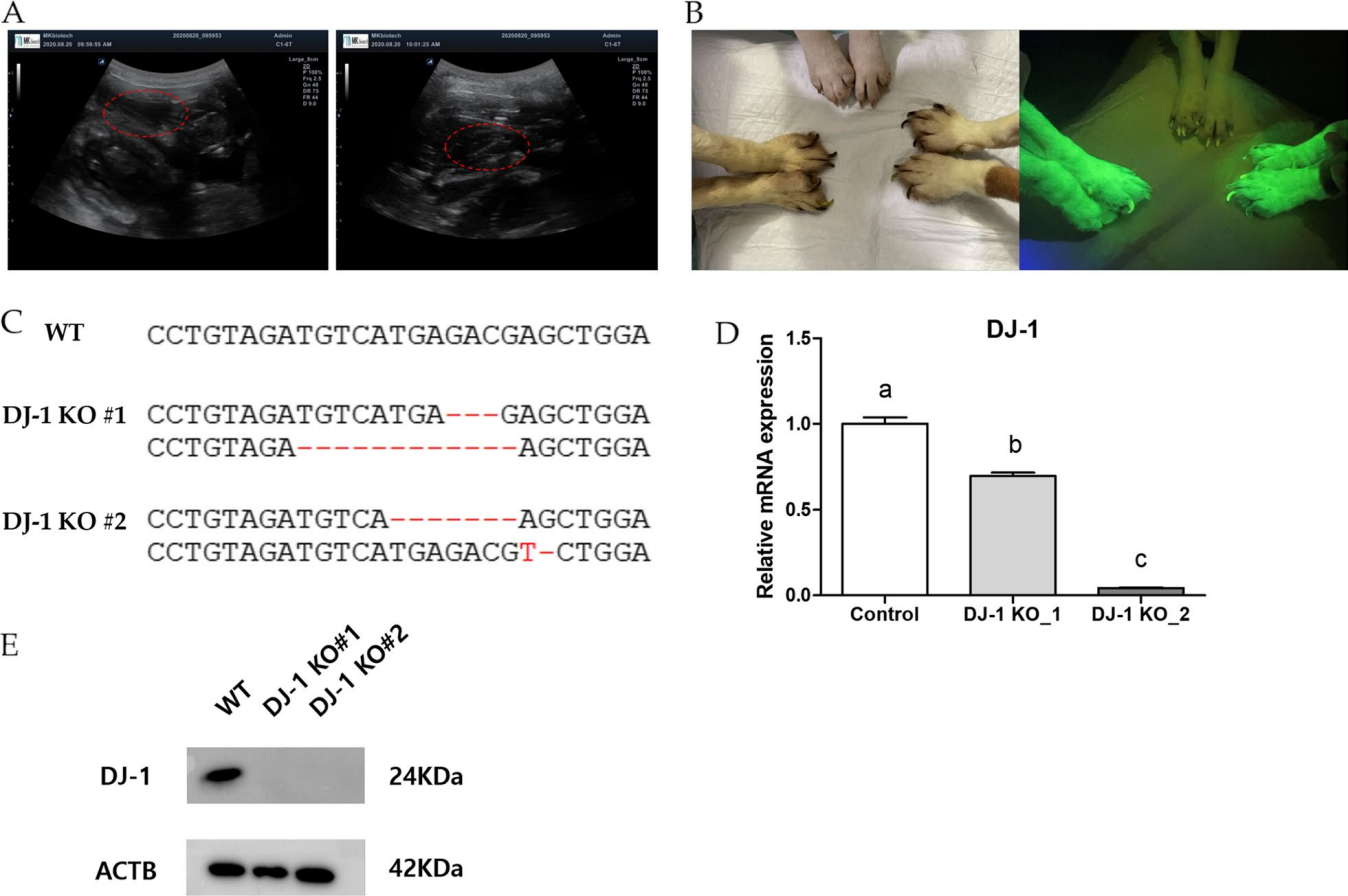
Genetic analysis of genome-edited dogs. **A** Pregnancy was diagnosed using ultrasound on day 45 after embryo transfer (fetuses circled in red). **B** Comparison of GFP expression in wild-type and DJ-1 knockout (KO) dogs. Image on the left was taken under bright field microscopy, and that on the right shows the expression of GFP. Wild-type dog is in the middle of the photo and DJ-1 KO#1 and DJ-1 KO#2 dogs are on the right and left sides, respectively. **C** Sequence analysis of the targeted locus in DJ-1 KO dogs. **D** Analysis of DJ-1 gene expression in DJ-1 KO dogs using real-time qRT-PCR. The X-axis represents the control, DJ-1 KO#1, and DJ-1 KO#2, respectively. The Y-axis represents the mRNA expression level of DJ-1 relative to the control (The data represent the mean values and standard deviations of five reaction replicates). **E** Western blot analysis of DJ-1 expression in DJ-1 KO dogs. ACTB was used to normalize the expression levels

We collected fibroblasts from a beagle dog fetus and established a DJ-1 KO cell line by transfection with the CRISPR-Cas9 vector, followed by neomycin selection for 14 days. We confirmed the expression of the CRISPR-Cas9 vector by evaluating the GFP expression (Fig. 1C, D). DJ-1 KO SCNT embryos were produced by fusing enucleated canine oocytes with DJ-1 KO cells. We transferred 68 SCNT embryos into six recipient dogs. Among them, one recipient was pregnant, producing two DJ-1-deficient offspring (Table 1 and Fig. 2A). The overall efficiency of CRISPR-Cas9-based genome-edited dog production using SCNT is 3.7%. This is similar to the efficiency reported in previous studies that used the microinjection method to produce genome-edited dogs (5.7% [19] or 3.1% [18]).

**Table 1:** Production of DJ-1 knock-out dogs by somatic cell nuclear transfer

No. of transferred SCNT embryos	No. of recipients	No. of pregnancy (pregnancies/recipients, %)	No. of offspring (births/transferred embryos, %)
68	6	1 (16.7)	2 (2.9)

We confirmed that both DJ-1-deficient dogs expressed EGFP using the selective reporter on the vector construct (Fig. 2B). The copy number and integrated site of the vector were analyzed from whole-genome sequencing results and are presented in Additional file 1: Table S1. Genome-edited mutations were analyzed by sequencing. Interestingly, DJ-1 KO #1 showed in-frame (− 3 bp/− 12 bp) mutations, whereas DJ-1 KO #2 showed out-of-frame (− 7 bp/− 1 and 1 substitution) mutations (Fig. 2C). In contrast to out-of-frame mutations that induce premature stop codons and completely disrupt the peptide structure, in-frame mutations delete only a few amino acids; thus, these mutations may not completely knock out gene function but may alter the activity or function of the gene product. Real-time quantitative reverse transcription polymerase chain reaction (qRT-PCR) results for fibroblasts obtained from DJ-1 KO dogs showed that DJ-1 expression was partially downregulated in DJ-1 KO #1 dog and completely repressed in the DJ-1 KO #2 dog (Fig. 2D, *p* < 0.0001). These two genotypes provide options for using these animals to further study the function of DJ-1. In addition, western blotting was performed to analyze the effects of DJ-1 KO at the protein level. As expected, the DJ-1 peptide was not detected in cells derived from DJ-1 KO dogs but was expressed in the wild-type control (Fig. 2E). All knockout dogs generated in the current study were healthy and did not show any abnormal phenotype until now (age: 14 months). Since DJ-1 related diseases, including Parkinson’s syndrome, are age-related, we are continuously monitoring the phenotype of knockout dogs for further studies (Additional files 2, 3).

One potential limitation of the current study is that we used an integrated CRISPR-Cas9 vector rather than CRISPR-Cas9 ribonucleoprotein or transient expression of CRISPR-Cas9 DNA to generate genome-edited dogs. Because of the low efficiency, high labor and cost of canine cloning procedures, we performed vector integration and antibiotic selection to prepare SCNT donor cells. However, the integrated CRISPR-Cas9 vector may induce high levels of off-target effects in the genome. The CRISPR-Cas9 system is active in dogs with DJ-1. Thus, there is a higher risk of off-target mutations mediated by CRISPR-Cas9 than during the transient expression of the CRISPR-Cas9 system. Therefore, we performed an off-target analysis in DJ-1 KO dogs. Through in situ analysis of the canine genome, we identified various potential off-target sites. We performed whole-genome sequencing of DJ-1 KO dogs and analyzed their genomic sequences at potential off-target sites. No off-target mutations were detected at any of the potential sites that we designed (Table 2). Our results suggest that the off-target effects of the CRISPR-Cas9 system on the canine genome were not severe. Further studies aimed at generating SCNT-derived genome-edited dogs using ribonucleoproteins or transient expression of CRISPR-Cas9 are required.

**Table 2:** In situ design of potential off-target locus

	Sequences	Chr	Position	Dir	Mismatch	Mutation (#1/#2)
On-target	GTAGATGTCATGAGACGAGCNGG	5	61577522	+	0	
Off-target 1	GTAGATGTgATGAcAgGAGCTGG	8	12929143	+	3	WT/WT
Off-target 2	cTAaATGTCATGAGAaGAGCAGG	30	14653215	+	3	WT/WT
Off-target 3	GTAGATGTCAgGAGAgGAGgGGG	7	39969232	+	3	WT/WT
Off-target 4	GTgGATGgCATGAGACGAGtGGG	17	53221459	−	3	WT/WT
Off-target 5	GTAGATGTgATGAaAtGAGCTGG	11	51558749	+	3	WT/WT
Off-target 6	aTAGATGTCATGAGAtGAGCTGG	24	32851089	−	2	WT/WT

Letters with lowercase indicate mismatches with on-target sequence

*Chr* chromosome number, *Dir* direction, *Mismatch* number of mismatches, *Mutation* sequence compared with WT

The SCNT method has several advantages as a platform to generate genome-edited dogs. Unlike the microinjection method used in previous studies, which involves direct injection of CRISPR-Cas9 into fertilized zygotes, breed and other genetic traits, except for the target gene, can be maintained if SCNT is performed to produce genome-edited dogs. This is particularly important in correcting pathogenic mutations in purebred dogs. Because breeding programs with a limited number of founder dogs are strictly selective, purebred dogs have a greater risk of genetic disorders than any other species [32]. Using genome-editing tools, SCNT-based production of pathogenic gene-corrected dogs is an excellent solution to this problem because it does not change the desired phenotype of a specific breed. In contrast, SCNT is useful for producing a group of genome-edited disease model dogs with the same genetic background, which will provide more reliable and stable data for researchers using animal models.

## Conclusion

We developed genome-edited dogs using SCNT and CRISPR-Cas9. DJ-1 KO dogs show partial or complete repression of target gene expression. This technology can be used in further studies to produce pathogenic gene-corrected or disease-modeling dogs.

## Methods

### Animals and ethics statement

The experimental procedures and methods used in this study were approved by the Animal Welfare and Ethics Office (CNU-01089) of Chungnam National University, Daejeon, and were performed in accordance with the Guide for the Care and Use of Laboratory Animals published by the IACUC of Chungnam National University and ARRIVE guidelines (https://arriveguidelines.org). Mixed female dogs (2–6 years of age) purchased from Honghwa Inc. (Nonsan, Republic of Korea) were used as oocyte donors and embryo transfer recipients. The dogs were housed in an approved breeding facility provided by Chungnam National University. Briefly, the dogs were housed in an environment with 12 h (07:00–19:00) of bright light and 12 h (19:00–07:00) of dark. The temperature of the breeding room was 22–23 °C with 50%–60% humidity. The dogs were housed in separate cages that were cleaned daily. Meals (Good Boy Jindo, Jeil pet food, Seoul, Republic of Korea) were served twice a day (09:00 and 17:00) and freshwater was provided freely.

### Establishment of canine fetal fibroblast

Canine fibroblasts were obtained from the fetus of an artificially inseminated beagle dog. Briefly, 28 days after artificial insemination, the dog was anesthetized with 6 mg/kg ketamine and 1 mg/kg xylazine intravenously and maintained with 2% isoflurane. After surgical removal of the fetus from the mother, the fetus was placed in a CO_2_ chamber for 10 min and all functions were allowed to cease. After the surgery, the dog was taken care of in the recovery room and pain was kept under control by administering analgesics and regular disinfection. After 10 days, the suture was removed and after confirming that the mother was stable, it was transferred to a conventional cage. To ensure that the fetuses were terminated, all other procedures were performed at least 30 min later. Each fetus was washed several times with phosphate-buffered saline, and the organs, limbs, and head were carefully removed. The remaining tissue was homogenized in phosphate-buffered saline with 0.5% fetal bovine serum (FBS) using a sterile BP blade No. 22 in a sterilized Petri dish containing the culture medium (DMEM). The chopped tissue was washed twice by centrifugation, placed in Dulbecco’s modified Eagle’s medium (Gibco, Grand Island, NY, USA) supplemented with 10% FBS (Gibco) and 1% penicillin/streptomycin (Gibco), and incubated at 5% CO_2_ at 39 °C. When the cells flowed out of the tissue, it was removed and cultured by replacing the medium. The cultured fibroblasts were stored at − 190 °C in cryopreservation solution containing 10% dimethyl sulfoxide and 90% FBS until use.

### Establishment of DJ-1 knockout canine fibroblasts

The target guide RNA (gRNA) for the CRISPR-Cas9 system was designed using CRISPR RGEN Tools (http://www.rgenome.net/). The selected gRNA sequence, 5′-GTAGATGTCATGAGACGAGC-3′ (Fig. 1A), was inserted into the PiggyBac-based commercially available CRISPR-Cas9 vector (VectorBuilder, USA). The vector construct also contained an EGFP/neomycin-resistance fusion gene for visualization and antibiotic selection (Fig. 1B). The CRISPR-Cas9 vector construct was co-transfected with the transposase vector (System Biosciences) into canine fibroblasts to establish the DJ-1 knockout cell line using TurboFect reagent (Thermo Fisher Scientific) following the manufacturer’s instructions. At 24 h after transfection, the fibroblasts were treated with 1 µg/mL neomycin for 14 days and then grown by maintaining antibiotics at 200 ng/mL. All fibroblasts were cultured in DMEM (Gibco) supplemented with 15% FBS (Gibco) and 1% penicillin/streptomycin (Gibco) at 38 °C and 5% CO_2_.

### Collection of in vivo matured oocytes

Mature oocytes were collected in vivo as previously described [33]. After confirming the time of estrus, blood was collected and progesterone levels in the blood were measured using VET Chroma (ANIVET Inc., Chuncheon, South Korea). Three days after ovulation, after the concentration of progesterone reached 4.0–7 ng/mL, mature oocytes were surgically collected. The dogs were anesthetized with 6 mg/kg ketamine and 1 mg/kg xylazine, and maintained with 2% isoflurane. After exposing the ovary and uterus, a 24G intravenous catheter was inserted into the oviductal lumen near the uterotubal junction, and the culture medium was allowed to flow to collect the mature oocytes. The culture medium was prepared by adding 2 mM NaHCO_3_, 1% penicillin/streptomycin, 0.5% bovine serum albumin, and 10% FBS to Medium 199 containing 25 mM HEPES.

### Production of DJ-1 KO dogs by SCNT

To produce DJ-1 KO dogs, SCNT followed by embryo transfer was performed as described in our previous report [33]. Briefly, in vivo matured canine oocytes with their first polar bodies were used for micromanipulation. The metaphase chromosomes were removed via oocyte aspiration. A single cell from the cultured genome-edited fibroblasts was transferred into the perivitelline space of an enucleated oocyte, and each donor cell-cytoplast couplet was fused with two pulses of direct current (24–26 V for 15 μs) using an electro-cell fusion apparatus (NepaGene, Chiba, Japan). The fused SCNT embryos were chemically activated by incubation in 10 μM calcium ionophore (Sigma, St. Louis, MO, USA) for 4 min and then in 1.9 mM 6-dimethylaminopurine (Sigma) for 3.5 h. Activated SCNT embryos were surgically transferred into the oviducts of estrus-synchronized surrogates on the same day as SCNT embryos. Thirty days after embryo transfer, pregnancy was confirmed by ultrasound scan, and cloned knockout dogs were safely delivered by cesarean section at full term.

### Analysis of on- and off-target mutations in DJ-1 KO dogs

PCR analysis followed by sequencing was performed to identify genome-edited on-target mutations in the cloned dogs. Total genomic DNA from founder dogs was isolated from the umbilical cord and used for PCR amplification using the primers F:5′-CTGCTGTGTTTTCATCTC-3′ and R:5′-AGTATCCAGGAAATTTAC-3′. PCR products were sequenced using a commercial vendor (Bioneer, Daejeon, Korea).

We identified potential off-target sites with two or three mismatches to sgRNA (Table 2) using an in situ design tool (Cas-OFFinder; http://www.rgenome.net/cas-offinder/). Whole-genome sequencing was performed by a commercial vendor (Bioneer), and sequences at potential off-target sites were analyzed.

### Analysis of DJ-1 expression in genome-edited dogs

Target gene expression was analyzed by real-time qRT-PCR. Primary fibroblasts were isolated from genome-edited dogs. The primary cell culture was performed under the same conditions as the SCNT donor cell culture described above. Total RNA was extracted from cultured primary cells and normal beagle cells as a control, and real-time qRT-PCR was performed to analyze the expression of DJ-1 in genome-edited cells. The primer sequences used for real-time qRT-PCR were β2-microbulin F:5′-CCAATGAGCAGGATGAGTT-3′ and R:5′-TTGTCTCGGTCCCACTTA-3′ and DJ-1 F:5′-GGACCTTATGACGTAGTGATT-3′ and R:5′-CTTTGCTTCCAAAACCTATTT-3′. The PCR conditions were as follows: 95 °C for 10 s, 52 °C for 30 s, and 72 °C for 30 s for 40 cycles.

### Immunoblot analysis

Proteins were extracted from cultured fibroblasts isolated from DJ1-KO #1 and DJ-1 KO #2 dogs. Total protein was quantified using bicinchoninic acid (Sigma) and 2 µg of each protein was used for western blot analysis. Anti-DJ-1 (Santa Cruz Biotechnology, Dallas, TX) was used as the primary antibody and anti-ACTB (ABclonal, Wuhan, China) was used as a control. Anti-mouse-horseradish peroxidase and anti-rabbit-horseradish peroxidase were used as the secondary antibodies. To detect the DJ-1 protein, bands were identified using an enhanced chemiluminescence solution (Bio-Rad, Hercules, CA, USA).

## Supplementary Information

**Additional file 1.** Supplementary data.

**Additional file 2.** Raw western blotting data for ACTB expression.

**Additional file 3.** Raw western blotting data for DJ-1 expression.

## Data Availability

The datasets generated and/or analyzed during the current study are not publicly available due to some data required for our further studies but are available from the corresponding author on reasonable request.
