# Supplementary material for: Generation of genome-edited dogs by somatic cell nuclear transfer
Source: BMC Biotechnol. 2022 Jul 13;22:19. doi: 10.1186/s12896-022-00749-3 (PMC9281017; doi:10.1186/s12896-022-00749-3)
Supplement: Supplementary file 1 — Additional file 1. Supplementary data. [file 12896_2022_749_MOESM1_ESM.docx]

***Supplementary data***

Generation of genome-edited dogs by somatic cell nuclear transfer

Dong-Ern Kim ^1^, Ji-Hye Lee ^1^, Kuk-Bin Ji ^1^, Kang-Sun Park ^2^, Tae-Young Kil ^3^, Okjae Koo ^4,^* and Min-Kyu Kim ^1,2,^*.

^1^ Laboratory of Animal Reproduction and Physiology, Department of Animal Science and Biotechnology, College of Agriculture and Life Science, Chungnam National University, 34134 Daejeon, Korea; [labmaster11@naver.com](mailto:labmaster11@naver.com)

^2^ MK biotech, 34134 Daejeon, Korea; [kminkyu@cnu.ac.kr](mailto:kminkyu@cnu.ac.kr)

^3^ Department of Social Welfare, Joongbu University, Geumsan 32713, Korea

^4^ ToolGen Inc., 08501 Seoul, Korea; [oj.koo@toolgen.com](mailto:oj.koo@toolgen.com)

* Correspondence

Okjae Koo:

ToolGen Inc., 08501 Seoul, Korea

[oj.koo@toolgen.com](mailto:oj.koo@toolgen.com); Tel.: +82-10-7445-7297

Min-Kyu Kim:

Laboratory of Animal Reproduction and Physiology, Department of Animal Science and Biotechnology, College of Agriculture and Life Science, Chungnam National University

[kminkyu@cnu.ac.kr](mailto:kminkyu@cnu.ac.kr); Tel.: +82-10-5208-1995

**Table S1 Copy number of CRISPR-Cas9 inserted into genome**

| **Dog ID** |  | **NO. of reads** | **Direction**^a^ | **Chr** | **Position** |
| --- | --- | --- | --- | --- | --- |
| KO#1 | Insert 1 | 11 | <- | 18 | 25147883 |
|  | Insert 2 | 13 | <- | 20 | 34906950 |
|  | Insert 3 | 11 | <- | 30 | 22470649 |
|  | Insert 4 | 13 | <- | 5 | 40276226 |
|  | Insert 5 | 10 | -> | 1 | 50851907 |
|  | Insert 6 | 10 | -> | 17 | 63125031 |
|  | Insert 7 | 10 | -> | 22 | 25901437 |
|  | Insert 8 | 10 | -> | 9 | 34690524 |
|  | Insert 9 | 10 | -> | X | 26026953 |
| KO#2 | Insert 1 | 15 | <- | 1 | 70904617 |
|  | Insert 2 | 10 | <- | 11 | 9921077 |
|  | Insert 3 | 12 | <- | 14 | 21345710 |
|  | Insert 4 | 10 | <- | 16 | 5612363 |
|  | Insert 5 | 10 | <- | 17 | 47081278 |
|  | Insert 6 | 10 | <- | 19 | 20325602 |
|  | Insert 7 | 21 | <- | 2 | 15471843 |
|  | Insert 8 | 10 | <- | 22 | 4264961 |
|  | Insert 9 | 10 | <- | 26 | 27577630 |
|  | Insert 10 | 16 | <- | 28 | 23736348 |
|  | Insert 11 | 18 | <- | 5 | 50686460 |
|  | Insert 12 | 10 | <- | 8 | 6310870 |

^a^ Direction indicates the direction of the inserted gene. -> indicates Forward of 5'->3', and <- indicates Reverse of 3'->5'.
