# Supplementary figures and images for: Generation of genome-edited dogs by somatic cell nuclear transfer
Source: BMC Biotechnol. 2022 Jul 13;22:19. doi: 10.1186/s12896-022-00749-3 (PMC9281017; doi:10.1186/s12896-022-00749-3)

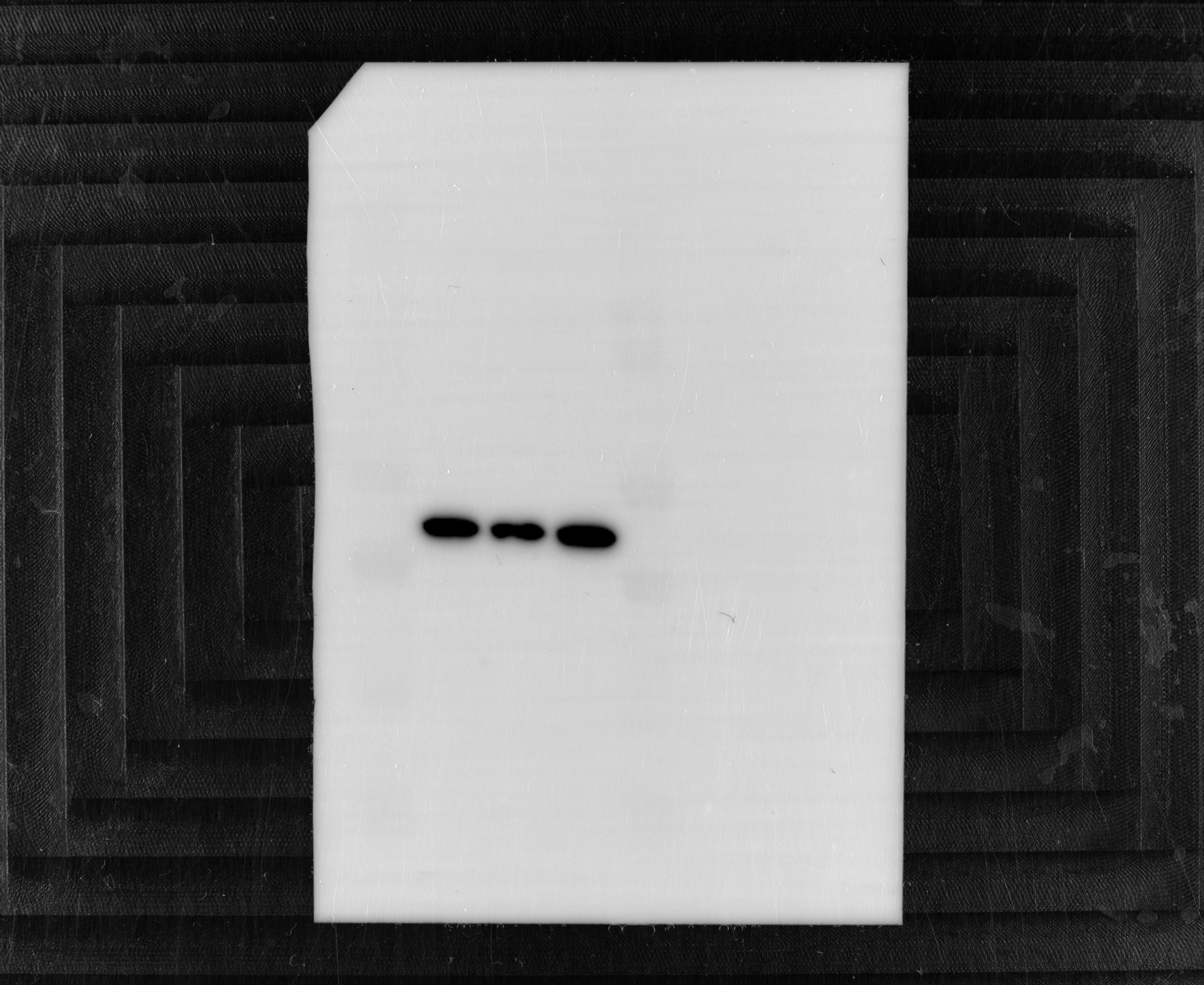

Supplement: Supplementary file 2 — Additional file 2. Raw western blotting data for ACTB expression. [file 12896_2022_749_MOESM2_ESM.tif]

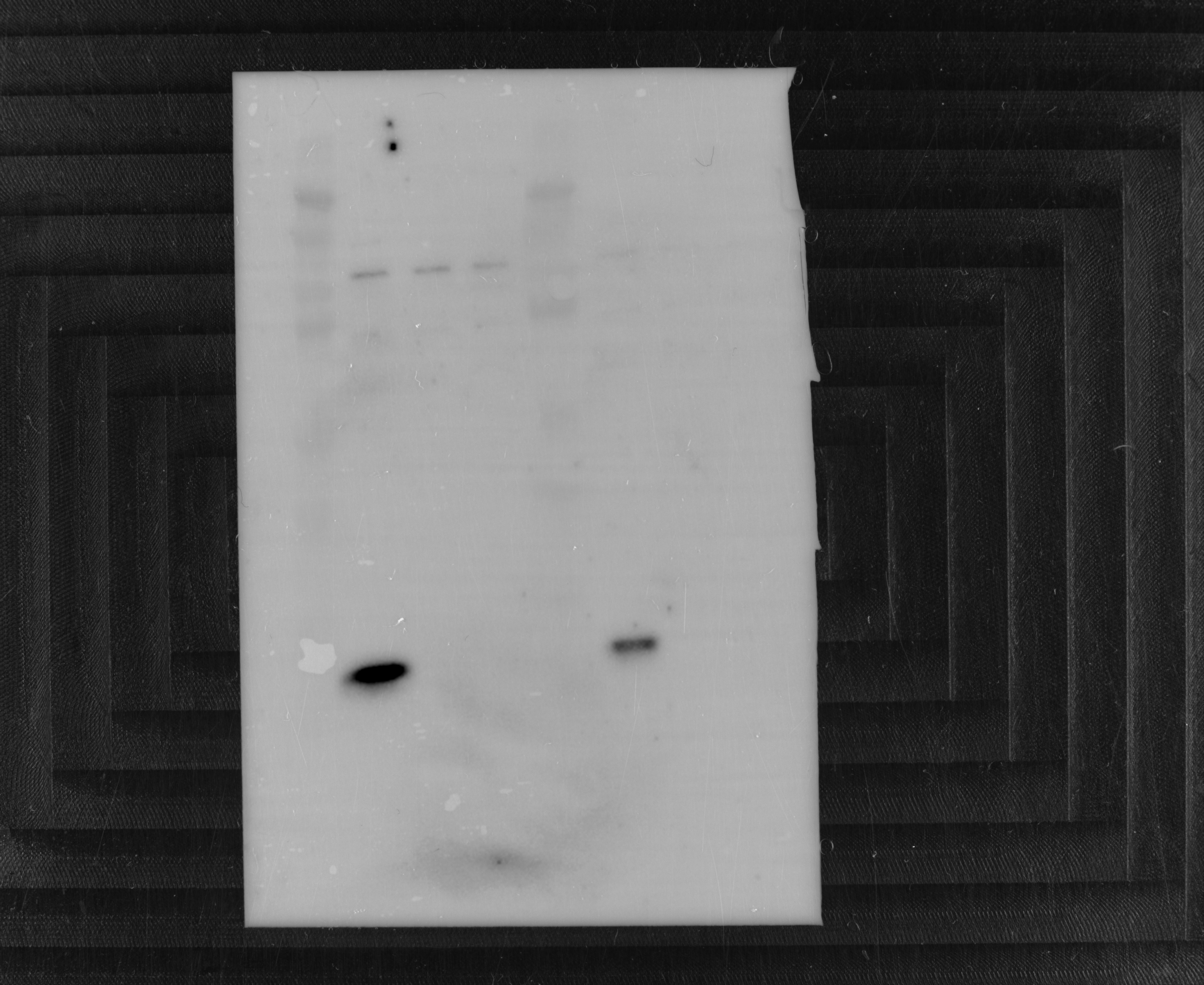

Supplement: Supplementary file 3 — Additional file 3. Raw western blotting data for DJ-1 expression. [file 12896_2022_749_MOESM3_ESM.tif]
